# *Andricus cydoniae* Giraud, 1859 Junior Synonym of *Cynips conifica* Hartig, 1843, as Experimentally Demonstrated (Hymenoptera: Cynipidae: Cynipini)

**DOI:** 10.3390/insects13020200

**Published:** 2022-02-15

**Authors:** Salvatore Sottile, Giuliano Cerasa, Bruno Massa, Gabriella Lo Verde

**Affiliations:** 1 Museo Civico di Lentate Sul Seveso, Via D. Aureggi 25, 20823 Lentate Sul Seveso, Italy; salvatore.sottile73@gmail.com; 2 Department of Agricultural, Food and Forest Sciences (SAAF), University of Palermo, Viale delle Scienze Bd. 5A, 90128 Palermo, Italy; bruno.massa@unipa.it (B.M.); gabriella.loverde@unipa.it (G.L.V.)

**Keywords:** oak gallwasp, heterogonic life cycle, biology, sexual generation, taxonomy, morphology, distribution, *Quercus*

## Abstract

**Simple Summary:**

Phytophagous members of the family Cynipidae induce a spectacular diversity of plant galls that are often complex in structure. Knowledge of the biology, life cycle, and life history of known cynipid species is largely fragmentary; gall wasps can exhibit an alternation of generations known as heterogony in which an all-female alternates with a bisexual generation. The unisexual generation produces eggs parthenogenetically, and these are usually inserted into a specific plant part. At the site of oviposition, galls are induced, within which a bisexual generation develops and later emerges. Emergent males and females mate, and females in turn induce galls from which the unisexual generation emerges. Females of the two generations may be morphologically dissimilar and may induce galls that differ greatly morphologically. Differences in the morphology of both wasps and galls between generations of the same species, coupled with incomplete knowledge of life cycles, have led to considerable taxonomic confusion. Alternating generations of numerous species have been described as separate species or even genera. Here, we demonstrate experimentally that two cynipid species, *Cynips conifica* (presently *Andricus conificus*) and *Andricus cydoniae*, which are morphologically different and produce very different galls on different host oaks, represent alternate generations of a single species.

**Abstract:**

We demonstrated the life cycle closure of *Cynips conifica* Hartig, 1843 (presently *Andricus conificus*), previously supposed on the basis of molecular data, and the identity of the sexual generation, through laboratory experiments. As a consequence, *Andricus cydoniae* Giraud, 1859 became a junior synonym of *A. conificus* (Hartig, 1843). We provide illustrations and a diagnosis for adults and galls, observations on biology, and information on distribution. Moreover, as sexual galls of *A. conificus* cannot be distinguished from those of *Andricus multiplicatus*, a detailed comparison between sexual galls and adults of these two species is reported.

## 1. Introduction

Approximately 1300 gall-forming wasp species have been described within the family Cynipidae [1,2,3], and among these 174 species are reported at present for the Italian fauna [4]. The most numerous genus associated with *Quercus* spp. is *Andricus* Hartig, 1840, which in Italy includes 70 species. Among them, heterogony has been demonstrated in 37 species [5,6,7,8,9,10,11].

The large number of observations conducted by Adler on several different species [12] and some recent studies in which the use of DNA sequencing allowed one to discover alternate generations (e.g., [11,13,14]) seem to show that the alternation of generations is the norm in the Cynipidae. In almost all remaining species for which only the sexual or asexual form is known, it is likely that alternate generation occurs but is yet to be described. This happens because in heterogonic gall wasps, the gall structure, phenology, and adult morphology differ between the asexual and sexual generations, even within the same species. Thus, it is possible that further studies on the biological cycle of these insects will lead to a reduction of the number of species to be considered as valid.

*Andricus conificus* was described by Hartig [15] as *Cynips conifica* with few morphological characters as was usual at that time. It was then transferred by Rohwer and Fagan [16] to *Adleria*, which was subsequently synonymised with *Andricus* (Benson in Marsden-Jones [17]).

*Andricus cydoniae* was described by Giraud [18], and in this case with few morphological characters. Moreover, descriptions of both species do not provide illustrations but give a good description of the galls induced.

Cook et al. [19] showed that *Andricus conificus* (Hartig, 1843), for which at present only the asexual generation is known, has the same DNA sequence for the cytb fragment found in wasps of the sexual generation of *Andricus cydoniae* Giraud, 1859 and supposed that these two gall-inducing cynipids could represent the alternate generations of a single species. Afterwards, Melika [20] reported the same hypothesis, without establishing their synonymy.

In the present study, we report results of laboratory assays and morphological identification, allowing to demonstrate that the sexual generation of *A. cydoniae* belongs to the previously described species *A. conificus.* Therefore, *Andricus cydoniae* Giraud, 1859 is here confirmed as junior synonym of *Andricus conificus* (Hartig, 1843).

We also provide information on the species distribution, illustrations, and diagnosis for adults and galls, highlighting the morphological differences between the asexual and sexual generation individuals and galls of this species and closest Western Palaearctic congeners.

## 2. Materials and Methods

### 2.1. Abbreviations Used in the Text

GCPC: Private collection of Giuliano Cerasa, Giuliana, Palermo, Italy
SSPC: Private collection of Salvatore Sottile, Cinisello Balsamo, Milan, Italy
MCLSS: Museo Civico Lentate Sul Seveso, Milan, Italy

### 2.2. Study Material Used in the Experiments

(1) Asexual females placed in contact chamber (Experiment 1):

1♀: ITALY: Lombardy, Pavia (Milano), loc. Orridi di Torrazza Coste Nature Park ex galls of *A. conificus* (ag) on *Quercus petraea*, 05.IX.2020, 44°57′03.6″ N 9°05′11.2″ E, 380 m, emerged 15.II.2021 (sample N. 4049), S. Sottile leg. (GCPC). 2♀: with the same label as the previous one but (SSPC).

(2) Sexual generation obtained from Experiment 1

3♀: ITALY: Lombardy, Cinisello Balsamo (Milano), ex galls of *A. cydoniae* (sex) in contact chamber on *Quercus cerris* (labelled as “Cerro A”), emerged 17.V.2021 (sample N. 4141), S. Sottile leg. (GCPC). 48♀: with the same label as the previous one but emerged 17-31.V.2021, (SSPC). 3♂: ITALY: Lombardy, Cinisello Balsamo (Milano), ex galls of *A. cydoniae* (sex) in contact chamber on *Quercus cerris* (labelled as “Cerro A”), emerged 17.V.2021 (sample N. 4140), S. Sottile leg. (GCPC). 25♂: with the same label as the previous one but emerged 17-31.V.2021, (SSPC& MCLSS).

(3) Sexual generation obtained from Experiment 1 and used in the Experiment 2:

4♀ & 3♂: ITALY: Lombardy, Cinisello Balsamo (Milano), ex galls of *A. cydoniae* (sex) in contact chamber on *Quercus cerris* (labelled as “Cerro A”), emerged 23.V.2021, S. Sottile leg. (SSPC).

### 2.3. Additional Material Examined for Morphological Diagnosis

5♀: ITALY: Lombardy, Pavia (Milano), loc. Orridi di Torrazza Coste Nature Park ex galls of *A. conificus* (ag) on *Quercus petraea*, 05.IX.2020, 44°57′03.6″ N 9°05′11.2″ E, 380 m, emerged 15-28.II.2021 (sample N.4048, N.4050), S. Sottile leg. (SSPC and MCLSS). 1♀: ITALY: Lazio, Monti Aurunci, Lenola (Latina) ex galls of *A. conificus* (ag) on *Quercus petraea*, 13.VIII.2020, 41°25′05.6″ N 13°28′47.8″ E, 400 m, emerged 25.II.2021 (sample N.4047), S. Sottile leg. (SSPC). 1♀: ITALY: Piemonte, Candelo (Novara), 45°32′12.5″ N 8°08′46.8″ E, 300 m, ex galls of *A. conificus* (ag) on *Quercus petraea*, 3.XI.2019, emerged 15.II.2021 (sample N. 4046), S. Sottile leg. (SSPC).

5♂ & 2♀: ITALY: Emilia-Romagna, Castelnovo né Monti (Reggio Emilia), ex galls of *A. cydoniae* (sex) on *Quercus cerris*, 28.V.2021, 44°25′38.1″ N 10°19′58.3″ E, 800 m, emerged 05.VI.2021 (sample N. 4161, 4162, 4163), S. Sottile leg. (SSPC). 29♀: ITALY: Liguria, Vobbia (Genova), Antola Natural Regional Park, ex galls of *A. cydoniae* (sex) on *Quercus cerris*, 27.V.2018, 44°35′10.1″ N 9°04′28.5″ E, 960 m, emerged 01.VI.2018 (sample N. 3624–3631), S. Sottile leg. (SSPC and MCLSS). 32♂: ITALY: Emilia-Romagna, Bagno di Romagna (Forlì-Cesena), lago Pontini ex galls on *Quercus cerris*, 09.VI.2018, 43°50′29.3″ N 12°00′12.5″ E, 770 m, emerged 30.VI.2018 (sample N. 3506–3514), S. Sottile leg. (SSPC & MCLSS). 2♀: ITALY: Sicily, Castelbuono (Palermo), loc. S. Guglielmo, ex galls of *A. multiplicatus* (sex) on *Quercus cerris*, 24.VI.2014, emerged 10-12.VII.2014 (sample N. 5447), G. Cerasa leg. (GCPC). 

### 2.4. Laboratory Assays

#### 2.4.1. Experiment 1

On 5 September 2020, fifteen galls of asexual generation of *A. conificus* were collected, near maturity, on branches of durmast oak (*Quercus petraea* (Matt.) Liebl.) at the Orridi di Torrazza Coste Nature Park, Pavia, Italy.

**Figure 1:**
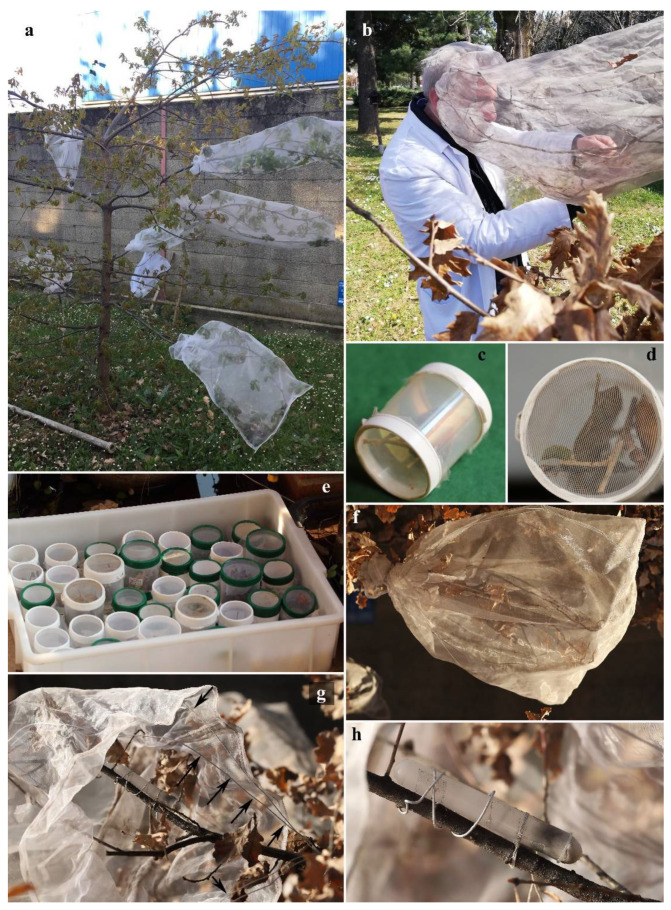
(**a**) Contact chambers on branches of young oak tree of *Quercus robur* L.; (**b**) S. Sottile during a periodic monitoring of the contact chamber; (**c**,**d**) details of plastic tubes in which the galls were kept; (**e**) plastic box with the bottom covered with soil and rotting oak leaves in which the galls are maintained for rearing in outdoor area; (**f**) contact chamber; (**g**) inner view of the contact chamber, the arrows show the wire framework maintains the tulle walls far from the branch; and (**h**) vial containing water-saturated cotton wool placed inside the contact chamber to provide water for the insects during egg-laying.

The twigs containing galls were maintained for about a month at room temperature, with their bottom end in water to preserve leaf turgidity, thus allowing for the maturation of the larvae. In October, they were transferred into 100 mL plastic tubes (Figure 1c,d), with tulle on the bottom and lid, which were placed in a plastic box with the bottom covered with soil and rotting turkey oak leaves (Figure 1e). The box containing galls was then placed outdoor in shady condition, checked every two weeks, and water was added when needed to maintain substrate moisture, until the emergence of gall-inducers, inquilines, parasitoids, and other inhabitants.

The first asexual females of gall-inducers emerged on 15.II.2021 and later at the end of February 2021. On 7 March 2021, three females (8 days-old) were placed into a “contact chamber” (Figure 1a,b,f,g) consisting of a tulle polyester bag (length 70 cm; width 40 cm; mesh size: mm 0.275 × 0.275; thread thickness: 50 µm) including a branch of a pot grown tree of *Quercus cerris* L. (11-year-old, labelled as “Cerro A”). To prevent oviposition by wild gall wasps, the branch chosen for the experiment was covered in the contact chamber about a month before the experiment started and the tulle bag was positioned to make sure that distance between tulle and the branch inside was at least 1 cm. The branch to be used in the experiment was chosen in a part of the tree, remaining always in shady conditions, to avoid a greenhouse effect inside the contact chamber, and at the same time with an exposure such as to ensure a good ventilation. A vial containing water-saturated cotton wool was placed inside the contact chamber to provide water for the insects during egg-laying (Figure 1h).

The three females inside the contact chamber laid eggs on the oak buds present in the branch and remained active until 14 March 2021. Afterwards, they were removed and mounted for subsequent morphological observations. The branch was left inside the tulle bag and checked every 2–3 days until the emergence of adult gall wasps, which started on 17 May.

#### 2.4.2. Experiment 2

On 24 May, 3♂ and 4♀ (7 days-old) obtained from Experiment 1 were transferred into 50 mL plastic jar with tulle on the lid in order to allow mating. In the following two days, several copulatory acts were observed. 

On 26 May, the insects were placed in a contact chamber (see above) (Figure 1a,b,f,g) that had been set up on a branch of young oak tree of *Quercus robur* L. (8-year-old, labelled as “Farnia 1F”) grown in a green peri-urban area (Gessate, Milano Province, Italy). Additionally, in this case the contact chamber was placed about a month before experiment, making sure that distance between tulle and the branch inside was at least 1 cm, to avoid the oviposition by wild wasps. Moreover, as galls of asexual form of *A. conificus* develop on adventitious buds in trunks and branches, the shoots were topped before being included in the contact chamber, to induce the adventitious buds development. A vial containing water-saturated cotton wool was placed inside the contact chamber to provide water for the insects during egg-laying (Figure 1h).

**Figure 2:**
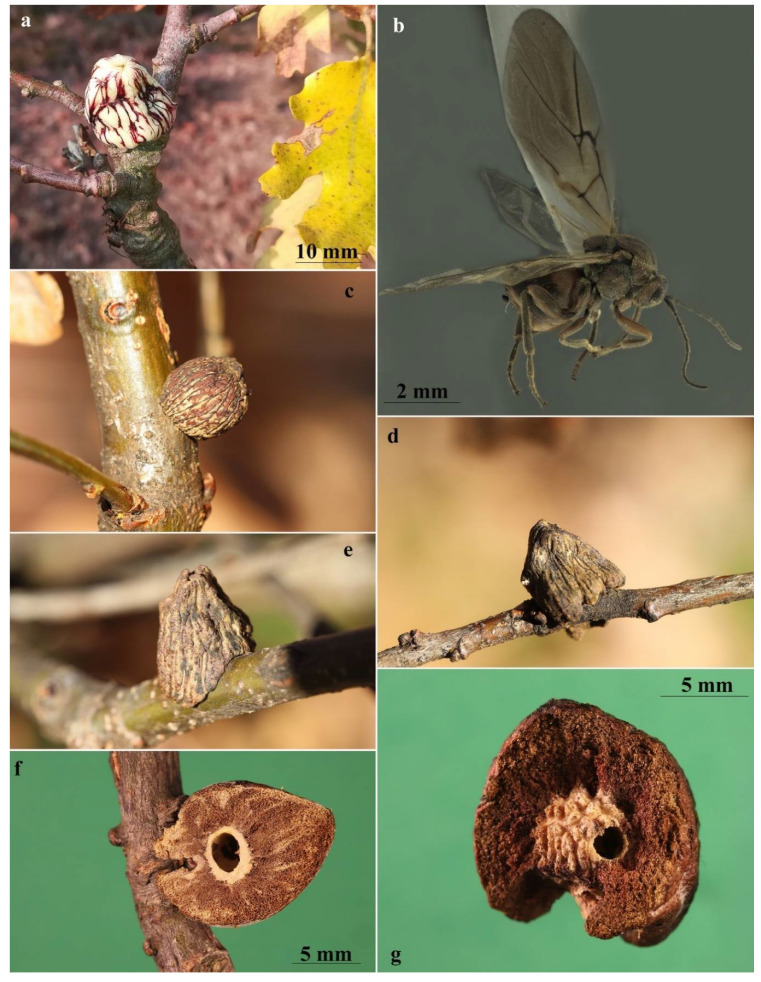
*Andricus conificus* asexual generation: (**a**) general appearance of the young gall on *Quercus petraea*; (**b**) habitus of adult (lateral view); (**c**–**e**) mature galls obtained from the second experiment; and (**f**) dissected gall, showing the larval chamber surrounded by spongy-suberous tissue. (**g**) This dissection shows the larval chamber surface covered in small protruding humps.

The adults in the contact chamber were checked daily, until male and female deaths occurred, after two days and after a week, respectively. Insects were then removed and mounted for subsequent morphological observations. The branch was left inside the tulle bag and checked every week for gall development (Figure 1b). No clear signs of gall development were recorded until mid-September, when, surprisingly, three developing *A. conificus* galls were found. After four weeks, the galls had already reached full development and turned a greyish colour (Figure 2c–e).

### 2.5. Morphological Study

Identification of adult wasps was performed using the keys and the morphological description provided by Melika [20] and Dalla Torre and Kieffer [21]. The original descriptions by Hartig [15] and Giraud [18] have been also considered. Insects were examined through a Wild-Heerbrugg M8 (Wild Heerbrugg, Heerbrugg, Switzerland) and a Kyowa Optical SD-2PL stereomicroscopes (Kyowa Optical, Tokyo, Japan) and with a Zeiss Universal Photomicroscope III compound microscope (Carl Zeiss, Oberkochen, Germany). Images were taken using a Leica DM series compound microscope (Leica, Benzheim, Germany) and a Leica DFC series mounted camera with Leica Application Suite software (LAS EZ 3.4.0, Leica, Switzerland). All insect photos were integrated using the freeware CombineZP [22] and processed in Adobe Photoshop CS4.

Galls were photographed with a Canon Eos 600D and Canon Eos 6D Mark II digital camera equipped with a Canon compact-macro lens EF 50 mm 1:2.5 and Canon macro lens EF 100 mm 1:2.8 L. (Canon Inc., Tokyo, Japan).

We follow the current terminology and abbreviations for morphological structures [20,23,24,25], antennal morphology and sensillar description [26], forewing venation [27], cuticular surface [28] and microsculpture [29]. Measurements and abbreviations used here include F1–F12, 1st and subsequent flagellomeres; POL (post-ocellar distance) is the distance between the inner margins of the posterior ocelli; OOL (ocellar-ocular distance) is the distance from the outer edge of a posterior ocellus to the inner margin of the compound eye; LOL is the distance between lateral and frontal ocelli; the diameter of a median or lateral ocellus is along its major axis; and tsa stands for transscutal articulation. Gula is the cranial area ventral to the posterior tentorial pits, defined laterally by the gular sulci, which converge in the postgenal suture. Acetabular carina is the area that is located medially on the epicnemial carina and posteriorly delimits the epicnemium; the width of the forewing radial cell is measured from the margin of the wing to the Rs vein; the petiole is the first metasomal tergite (T1); metasomal tergite 2 (T2) is the first obvious tergite; T3–T9 indicate subsequent tergites; and Ts1–Ts5 indicate first and subsequent tarsomeres.

Most of the anatomical terms used can be found in the Hymenoptera Anatomy Ontology (HAO) [30,31]. Most of the definitions can be also found at http://glossary.hymao.org (accessed on 20 September 2021).

## 3. Results

### 3.1. Laboratory Assays

After removing the gall wasp females, the development of the branch of “Cerro A” inside the contact chamber followed its natural spring progression until the second half of April, when several newly formed leaves appeared withered and did not develop regularly. On 2 May 2021, about 20 globular clusters of galls were observed, which continued to grow until maturity. Few galls reached 1.8 cm in diameter size, while most of them remained smaller than 1.5 cm at maturity, probably due to the fact that the host plant was a pot grown oak.

In the second half of May, the gall-inducers started to emerge, initially only males and then, from 21 May, both females and males with a peak on 26 May; the adults’ emergence was recorded until 5 June 2021. A total of 51 females and 28 males that emerged from the galls were identified as *A. cydoniae*, on the basis of morphological characters.

Therefore, according to the International Code of Zoological Nomenclature (ICZN) [32], a new synonymy is here established: *Andricus cydoniae* Giraud, 1859 as junior synonym of *A. conificus* (Hartig, 1843).

### 3.2. Gall

The asexual galls (Figure 2a,c–g) develop on the buds of thicker branches and on the main trunk to which they are attached by a thin stalk. Young galls are whitish with thin longitudinal red-purplish or brown veins from base to tip, covered with stellate hairs that fall at maturity when the galls turn darker and assume a suberous consistency. The galls are monolocular, about 10–15 mm high, subconical in shape with obtuse apex, and have a well-differentiated central larval chamber surrounded by spongy-suberous tissue (Figure 2f). The larval chamber consists of very compact woody tissue with a surface covered in small protruding humps (Figure 2g).

**Figure 3:**
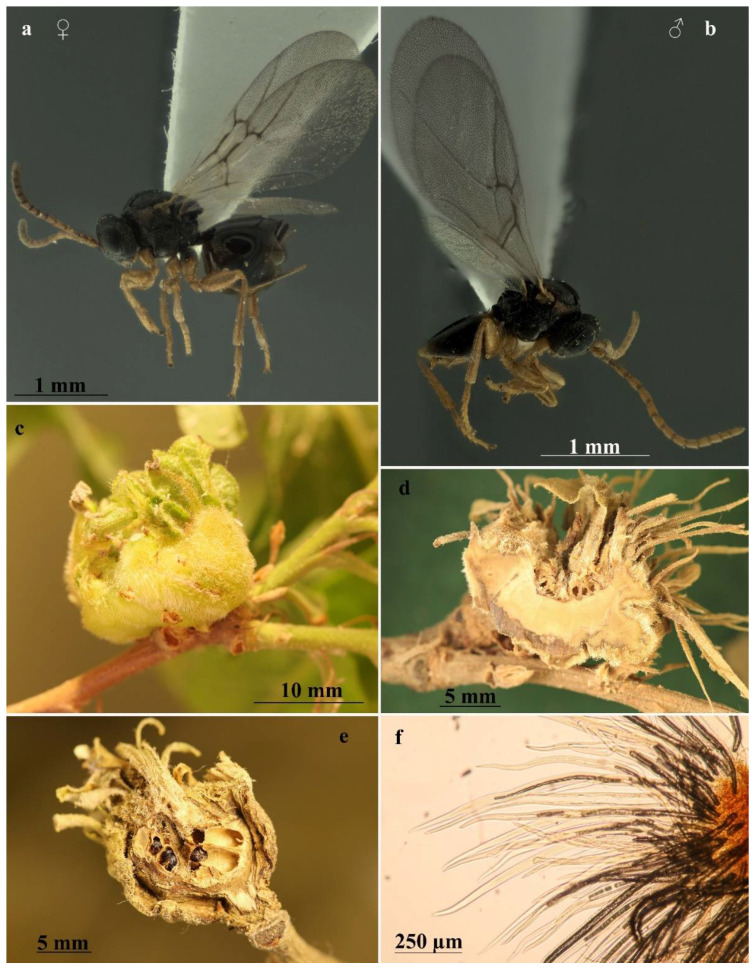
*Andricus conificus* sexual generation: (**a**,**b**) habitus, female and male (lateral view); (**c**) general appearance of gall; (**d**) dissected gall, showing the layer of compact vegetative tissue, which takes the shape of ‘open’ thalamus; (**e**) dissected gall, showing the layer of compact vegetative tissue, which takes the shape of ‘closed’ thalamus and the egg-shaped larval chambers; and (**f**) histological preparation showing the single-cell hairs.

The galls of the sexual generation (Figure 3c–f) consist of a hypertrophic degeneration of the terminal or lateral shoot buds; these buds are transformed into an ovoid or subspherical swelling with a diameter that can vary from 10 to 40 mm (often the galls are coalescent, forming large conglomerates and reaching sizes greater than 50 mm); they are light-green when young (Figure 3c) and dark-green and then brown when mature, bearing more or less deformed or normal leaves on the upper part.

The typical structure of the gall consists of a layer of compact vegetative tissue, 3 to 10 mm thick, which takes the shape of a more or less concave thalamus. This woody ‘thalamus’, sometimes ‘closed’ like a ceramic pot (Figure 3e), in other cases ‘open’ like a plate (Figure 3d), provides support and protection for the larval chambers (5 to 20) that develop above (Figure 3e). The surface of the gall ‘thalamus’ is externally covered with soft hairs that confer a velvety, silvery appearance to the gall. On the thalamus, curled, rakish leaves and twigs develop, and in some of these, larval cells can be ‘dragged along’ during development; these metamorphosed leaves wither as the galls mature and after adult emergence the galls may remain on the plant for some years. The inner part of the gall is covered with a dense layer of white single-celled hairs (Figure 3f) that also extends to the larval chambers, which are egg-shaped (Figure 3d,e) with enlarged base and apical extension more or less pointed and slightly curved, measuring 3.3–4.5 mm in height × 1.8–2.8 mm in width measured at 1/3 from the base. They develop cohesively, with each embedded in a socket of the supporting thalamus tissue like teeth in the gums (Figure 3d). Sometimes the constipated proximity of the chambers alters their egg shape to rectangular parallelepiped with rounded corners. When the gall thalamus is “opened”, the larval chambers are visible from above. In the inner part of the socket, the surface is coated with dense coverage of white hollow single-cell hairs (Figure 3f); these structures in a less dense form coat the larval cells with a distinct supporting cell layer. Whitish hairs are longest at the base of the larval chamber and become shorter at the apex. The inner layer of the larval chamber is thin and hard, composed of sub-rectangular cells arranged longitudinally to the length of the chamber; the emergence hole is in the apical or sub-apical part.

### 3.3. Similar Galls

Based on gall characteristics and host plant information, numerous gall-inducing insects can be identified to the species level. However, some exceptions have recently been reported in gall wasps and gall midges. For example, two congeneric gall midges, *Asphondylia gennadii* (Marchal, 1904) and *Asphondylia capsicicola* Uechi, Yukawa et Tokuda, 2016, induce the same kind of galls on the same plant organ and host plant species, but the gall midges themselves can be distinguished from each other based on pupal morphology and molecular differences [33]. With regard to the gall wasps, the sexual generation gall of a species recently described, *Latuspina jinzhaiensis* Abe, Ide, Su, et Zhu, 2021, is indistinguishable from that produced by *L. abemakiphila* Ide et Abe, 2021, which is induced on leaves of the same oak species in Japan [34].

**Figure 4:**
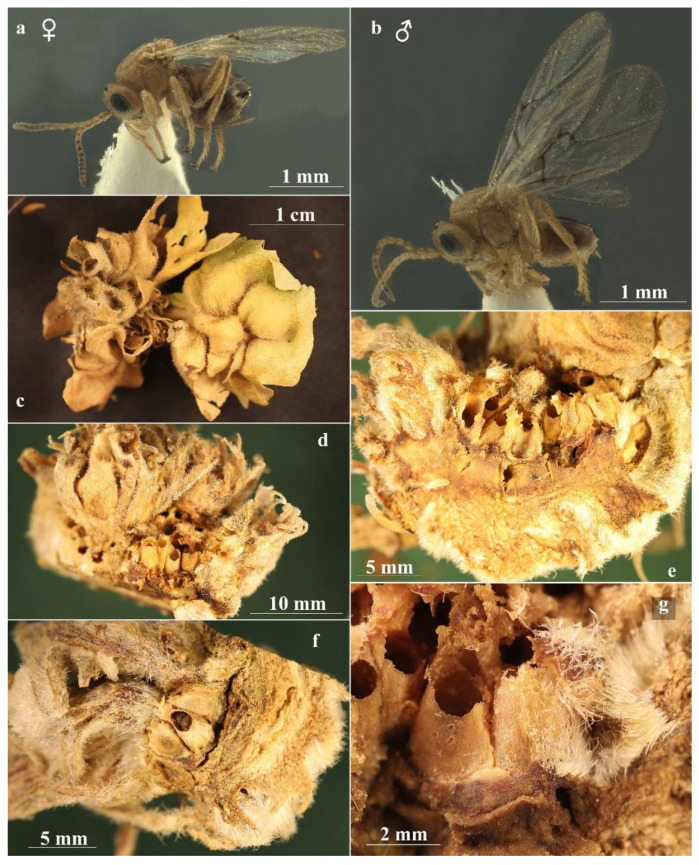
*Andricus multiplicatus* sexual generation: (**a**,**b**) habitus, female and male (lateral view); (**c**) general appearance of gall; (**d**–**f**) dissected gall, showing the egg-shaped larval chambers embedded in a socket of the supporting thalamus tissue; and (**g**) magnification of the larval chambers.

In our case, sexual generation galls of *A. conificus* are very similar only to those of *A. multiplicatus* (Figure 4c–g), and both develop on the same host plants. Melika [20] observed that the two galls can be confused and added that the galls of sexual generation of *A. conificus* are genuinely multilocular, with many larval chambers inside a solid mass of tissue, while those of *A. multiplicatus* are an aggregation of distinct galls.

The gall of *A. conificus* sexual generation is generally described as a concave ‘closed’ structure with an apical opening, in which are enclosed the larval chambers; however, we also found galls with ‘open’ thalamus in which the larval chambers are visible from above. This ‘open’ thalamus conformation has been described for *A. multiplicatus,* but we have also found closed galls for this species. In conclusion, we did not find any macro- or micro-morphological characters to distinguish the two galls with absolute certainty. Moreover, all the characters show variability in both species; therefore, we consider that it is impossible to identify the species only from the gall morphology. On the other hand, species identification through adults is relatively easy on the basis of morphological differences listed in Table 1. 

### 3.4. Diagnosis of the Asexual Form

**Figure 5:**
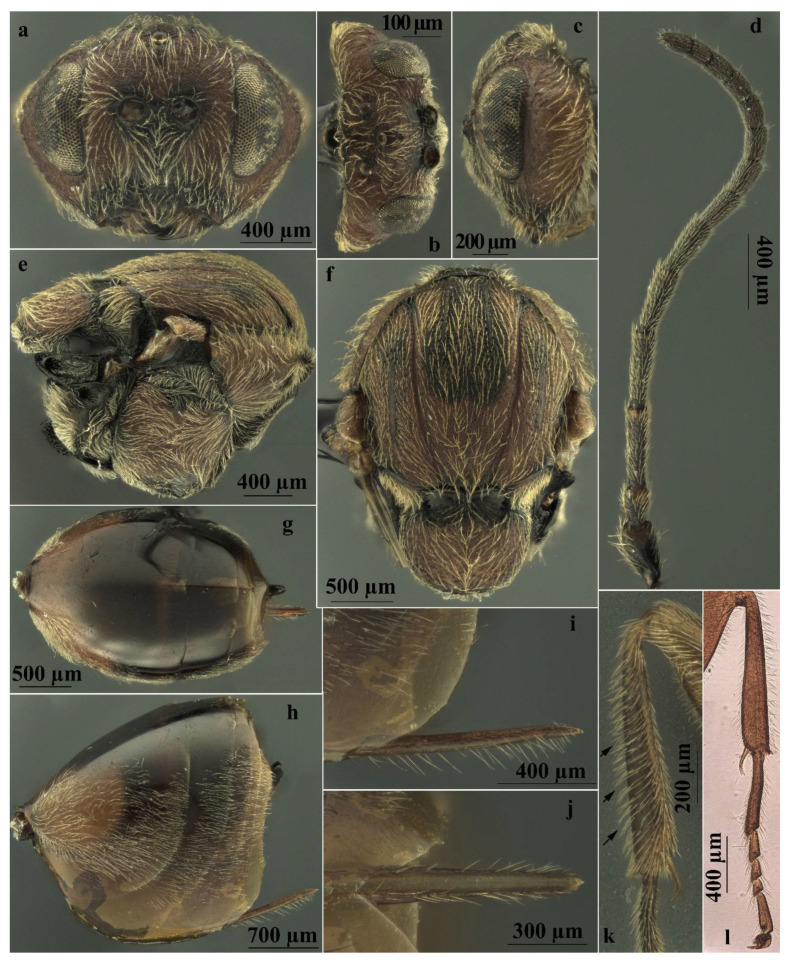
*Andricus conificus* asexual female: (**a**) head, (front view); (**b**) head (dorsal view); (**c**) head (lateral view); (**d**) antenna; (**e**) mesosoma (lateral view); (**f**) mesosoma (dorsal view); (**g**) metasoma (dorsal view); (**h**) metasoma (lateral view); (**i**) ventral spine of hypopygium (lateral view); (**j**) ventral spine of hypopygium, ventral view; (**k**) foretibia (the arrows show the long oblique setae on the anterior surface); and (**l**) foreleg on microscope slide.

Asexual females of *A. conificus* belong to “*Adleria*-non *kollari*” group, a large group of 13 *Andricus* species [35], with the anterior surface of foretibia bearing long oblique setae (Figure 5k,l); antenna 14-segmented (rarely 13 or 15) (Figure 5d); the mesoscutum coriaceous, without punctures (Figure 5f); and all metasomal tergites with dense white setae laterally (Figure 5h) and the prominent part of the ventral spine of the hypopygium needle-like and very long [35]. 

More specifically, in *A. conificus* (Figure 2b and Figure 5a–l) the prominent part of ventral spine of hypopygium is very long and slender, 6.25–7.0 times as long as broad in ventral view, with relatively short setae (Figure 5h–j). It closely resembles *A. truncicolus*; however, in *A. conificus* the body is reddish brown with black marks between notauli (Figure 2b and Figure 5e,f); the head is less rounded in front view, and the gena is broader than the compound eye for the entire height of the head (Figure 5a), while in *A. truncicolus* the body is blackish brown and the head is more rounded in front view and the gena is broader than the compound eye only behind and ventrally.

*Andricus conificus* resembles *A. synophri* Pujade-Villar, Tavakoli and Melika, 2015, from which it differs in having the body colour reddish brown, the body length around 4.0 mm, F1 longer than F2 (Figure 5d), and the metasomal tergites without micropunctures (Figure 5g,h), while *A. synophri* is smaller in size, around 3.0 mm, and has F1 slightly shorter than F2, micropunctures on the metasomal tergites, and a black body. 

### 3.5. Diagnosis of the Sexual Form

**Figure 6:**
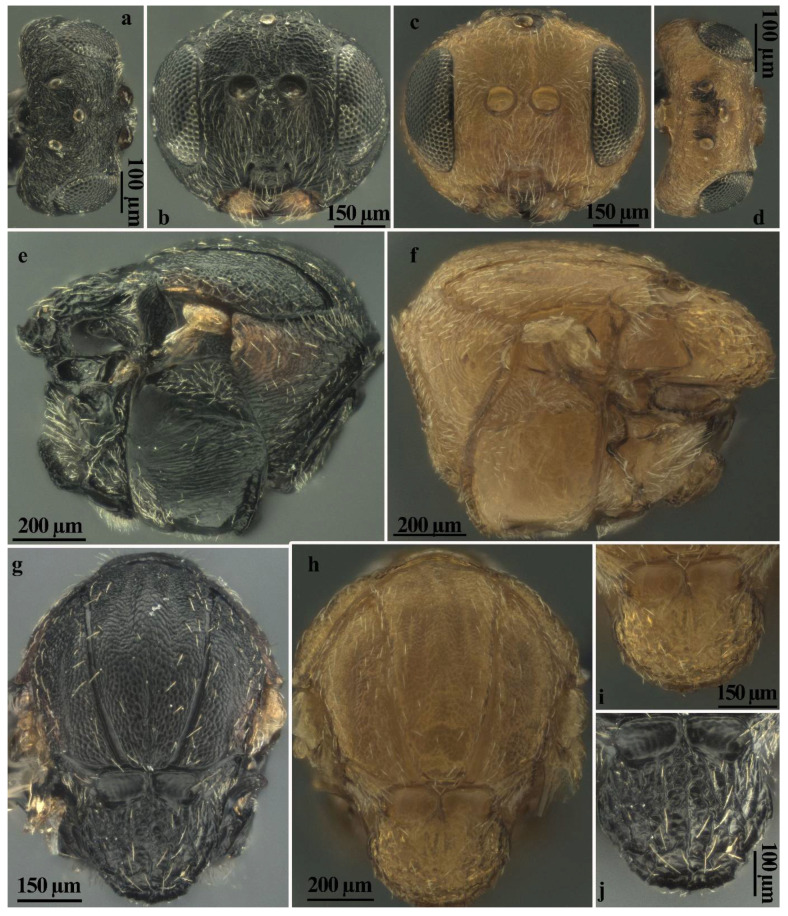
Comparison between females of sexual generation of *Andricus conificus* (**a**,**b**,**e**,**g**,**j**) and *A. multiplicatus* (**c**,**d**,**f**,**h**,**i**): (**a**,**d**) head (dorsal view); (**b**,**c**) head, (front view); (**e**,**f**) mesosoma (lateral view); (**g**,**h**) mesosoma (dorsal view); and (**i**,**j**) mesoscutellum (dorsal view).

**Figure 7:**
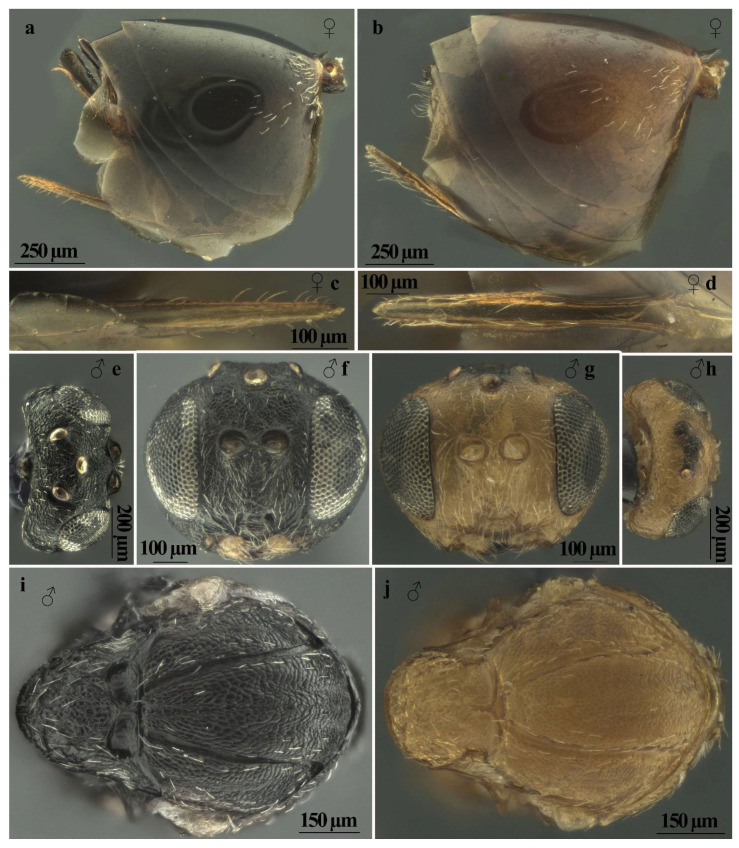
Comparison between females of sexual generation of *Andricus conificus* (**a**,**c**) and *A. multiplicatus* (**b**,**d**): (**a**,**b**) metasoma (lateral view); (**c**,**d**) ventral spine of hypopygium (ventral view); (**e**–**j**) comparison between males of sexual generation of *Andricus conificus* (**e**,**f**,**i**) and *A. multiplicatus* (**g**,**h**,**j**): (**e**,**h)** head (dorsal view); (**f**,**g**) head (front view); and (**i**,**j**) mesosoma (lateral view).

**Figure 8:**
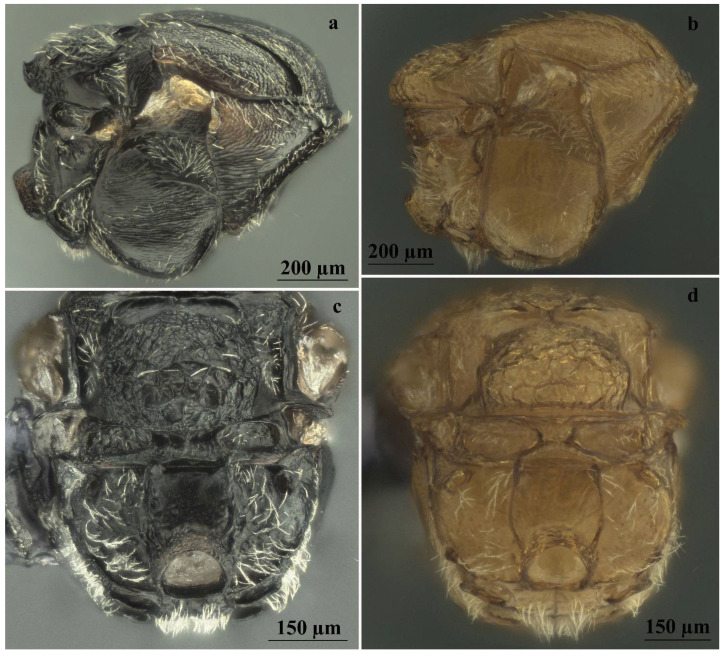
Comparison between males of sexual generation of *Andricus conificus* (**a**,**c**) and *A. multiplicatus* (**b**,**d**): (**a**,**b**) mesosoma (lateral view); (**c**,**d**) metascutellum and propodeum (posteroventral view).

*Andricus conificus* sexual form belongs to the group of species with a transversely striate mesopleuron (Figure 6e). Most closely resemble *A. cryptobius*. In sexual females (Figure 3a, Figure 6a,b,e,g,j and Figure 7a,c) with rounded mesoscutellum, the disk of the scutellum is dull rugose along sides, with much smaller units in the centre of the disk (Figure 6j); in males (Figure 3b, Figure 7e,f,i and Figure 8a,c), POL is at least 2.0 times as long as length of lateral ocellus (Figure 7e), and ocelli are much smaller, while in *A. cryptobius,* sexual female has slightly elongated mesoscutellum, the disk of scutellum is uniformly dull rugose, and main strong rugae are directed longitudinally (appearing parallel at low magnification). In males, POL is less than 2.0 times as long as the length of the lateral ocellus, and ocelli are large. 

### 3.6. Biology and Host Plant

This species has a heteroecic cycle; the galls of sexual generation begin to develop on *Cerris* section oak in early April and mature in May, and the adults emerge from the second half of May to the first half of June. The asexual galls begin development on *Quercus* section oaks in early summer, reach maturity in late August and September, and the adults emerge in February-March of the following year or in the second-year, as demonstrated for the first time by our sampling/emerging data (sample N. 4046), spending one year in diapause. The diapause is common in Cynipidae, and a high proportion of the asexual generation larvae of several species of cynipids shows diapause for periods ranging from 1 to, less frequently, 8 years [1].

### 3.7. Distribution

The species is widely distributed in the Western Palaearctic region: Austria, France, Bulgaria, Hungary, Romania, Kosovo, Poland, Greece, Ukraine (Transcarpathian Region only) [20], Switzerland [36], Serbia [37], Croatia [38] (including Cres-Lošinj Archipelago [39]), Slovakia [40], and Turkey [41,42]. Concerning Italy, it is reported for the northern and southern regions, including Sicily [43]. Regarding Sicily, records of *A. conificus* should be considered doubtful, as no record of *A. conificus* (ag) is known from literature, and it is considered impossible to distinguish the galls of *A. conificus* (sex) from those of *A. multiplicatus*.

## 4. Discussion

The “life cycle closure”, i.e., the process of determining the alternate generation of a heterogonic species or synonymizing two previously described univoltine species [5,44,45], can be problematic as the adults and gall morphology of both generations can differ markedly. Moreover, rearing experiments are often difficult, time-consuming, and do not always lead to positive results. Nevertheless, they can be considered essential to assess life cycle closure [8,9,46,47,48,49].

On the other hand, molecular methods may allow for pairing of currently unmatched sexual and asexual generations into a single species lifecycle, given that both generations within a species have identical (or nearly so) DNA sequences [11,13,14]; the two approaches are complementary and mutually reinforcing.

The results of the present study and our recent studies (e.g., [50,51]) show that much remains to be revealed about Cynipidae, even in the Western Palaearctic, where they are considered a relatively well studied family. Further studies will probably lead to a reduction of the number of valid species as a result of life cycle closure, as sexual and parthenogenetic generations of many species still remain unpaired. However, other new species remain to be described, and many biological aspects remain to be investigated.

## Figures and Tables

**Table 1:** Morphological differences between sexual form of *A. conificus* (=*A. cydoniae*) and *A. multiplicatus*.

**Features**	***A. conificus* ♀ (=*A. cydoniae*)**	***A. multiplicatus* ♀**
Body color	Mostly dark brown to black, with yellow legs, except for proximal part of hind coxae being dark brown (Figure 3a)	Mostly reddish yellow, legs slightly lighter than body (Figure 4a)
Head sculpture	Frons, vertex, and occiput reticulate (Figure 6a,b)	Frons, vertex, and occiput uniformly coriaceous (Figure 6c,d)
Striae on mesopleuron	With very marked striae (Figure 6e)	With or without very indistinct striae (Figure 6f)
Sculpture, shape, and size of mesoscutellum	As long as is broad; uniformly strongly areolate-rugose with distinct mainly longitudinal sharp rugae with emarginate posterior margin (Figure 6e,j)	Broader than long; reticulate rugose around its limits, more delicate in the central part of disk with unemarginate posterior margin (Figure 6f,i)
Shape of scutellar foveae	Scutellar foveae subtriangular well-delimited posteriorly (Figure 6g,j)	Subrectangular not or very slightly delimited posteriorly (Figure 6h,i)
Mesoscutum sculpture	Deeply colliculate (Figure 6g)	Shallowly colliculate (Figure 6h)
White setae on prominent part of ventral spine of hypopygium	Very few, short (approximately as long as the median diameter of the hypopygium in lateral view), erect, and not extending behind apex of ventral spine (Figure 7a, c)	Few, long (about one and a half times the median diameter of the hypopygium in lateral view), curved, and slightly extending behind apex of spine (Figure 7b,d)
**Features**	***A. conificus* ♂ (=*A. cydoniae*)**	***A. multiplicatus* ♂**
Body color	Mostly dark brown to black, with yellow legs, except for proximal part of hind coxae being dark brown (Figure 3b)	Mostly reddish yellow, legs slightly lighter than body (Figure 4b)
Ratio of diameter of torulus (including rims) to eye-torulus distance	Nearly 1.6 times eye-torulus distance (Figure 7f)	Equal to eye-torulus distance (Figure 7g)
Ratio of eye-torulus distance to distance between toruli	Nearly 1.4 times as large as distance between toruli (Figure 7f)	Nearly 3.0 times as large as distance between toruli (Figure 7g)
Frons and vertex sculpture	Rugose (Figure 7e,f)	Coriaceous (Figure 7g,h)
Shape of scutellar foveae	Subtriangular well-delimited posteriorly (Figure 7i)	Subrectangular Not or very slightly delimited posteriorly (Figure 7j)
Sculpture, shape, and size of mesoscutellum	Around its limits, strongly reticulate rugose; more delicate or colliculate in the central part of disk, with emarginate posterior margin (Figure 7i)	Around its limits, reticulate rugose; more delicate or coriaceous in the central part of disk, with unemarginate posterior margin (Figure 7j)
Mesoscutum sculpture	Deeply colliculate (Figure 7i)	Shallowly colliculate (Figure 7j)
Striae on mesopleuron	With very marked striae (Figure 8a)	With or without very indistinct striae (Figure 8b)
Ratio of breadth to height of metascutellum	More than 2.0 (Figure 8c)	Less than 1.5 (Figure 8d)

## Data Availability

The data presented in this study are available in article.
